# Terahertz Spin-Conductance Spectroscopy: Probing Coherent and Incoherent Ultrafast Spin Tunneling

**DOI:** 10.1021/acs.nanolett.4c00498

**Published:** 2024-06-21

**Authors:** Reza Rouzegar, Mohamed Amine Wahada, Alexander L. Chekhov, Wolfgang Hoppe, Genaro Bierhance, Jiří Jechumtál, Lukáš Nádvorník, Martin Wolf, Tom S. Seifert, Stuart S. P. Parkin, Georg Woltersdorf, Piet W. Brouwer, Tobias Kampfrath

**Affiliations:** † Department of Physics, Freie Universität Berlin, 14195 Berlin, Germany; ‡ Department of Physical Chemistry, Fritz Haber Institute of the Max Planck Society, 14195 Berlin, Germany; § Max Planck Institute for Microstructure Physics, Weinberg 2, 06120 Halle, Germany; ∥ Institut für Physik, Martin-Luther-Universität Halle, 06120 Halle, Germany; ⊥ Faculty of Mathematics and Physics, Charles University, Ke Karlovu 3, 121 16 Prague, Czech Republic

**Keywords:** Terahertz spintronics, spin conductance, terahertz spectroscopy, MgO tunnel junctions, coherent tunneling, incoherent resonant tunneling

## Abstract

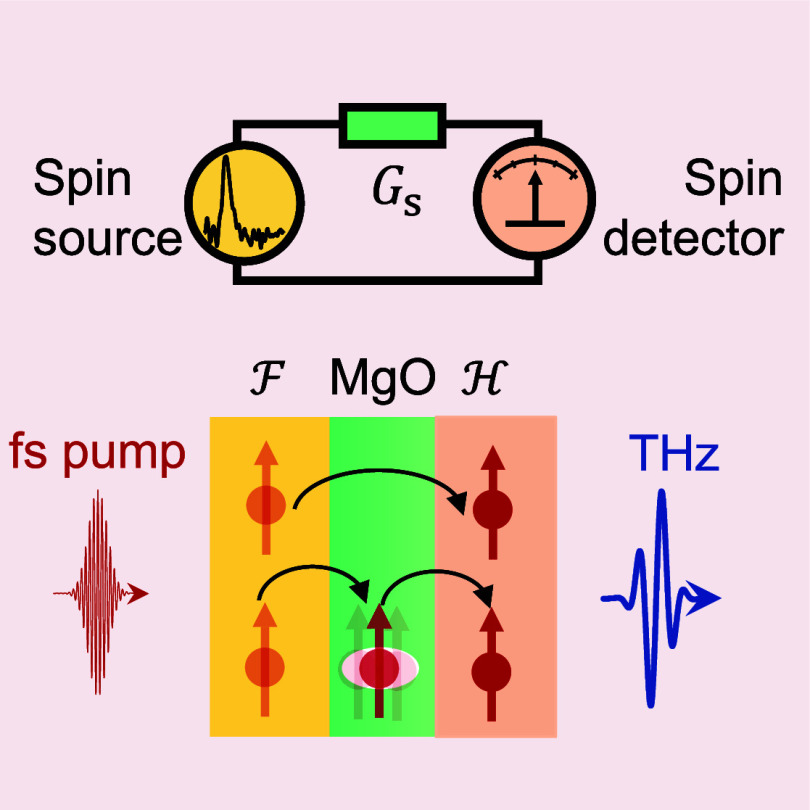

Thin-film stacks | consisting of a ferromagnetic-metal layer  and a heavy-metal layer  are spintronic model systems. Here, we present a method to measure the ultrabroadband spin conductance across a layer  between  and  at terahertz frequencies, which are the natural frequencies of spin-transport dynamics. We apply our approach to MgO tunneling barriers with thickness *d* = 0-6 Å. In the time domain, the spin conductance *G*_s_ has two components. An instantaneous feature arises from processes like coherent spin tunneling. Remarkably, a longer-lived component is a hallmark of incoherent resonant spin tunneling mediated by MgO defect states, because its relaxation time grows monotonically with *d* to as much as 270 fs at *d* = 6.0 Å. Our results are in full agreement with an analytical model. They indicate that terahertz spin-conductance spectroscopy will yield new and relevant insights into ultrafast spin transport in a wide range of spintronic nanostructures.

## Motivation

Transport of spin angular momentum by electrons is of central importance for future spintronic devices. To keep pace with other information carriers such as electronic charges in field-effect transistors^1^ and photons in optical fibers,^2^ spin transport needs to be pushed to terahertz (THz) bandwidth and, thus, femtosecond time scales. Promising applications of ultrafast spin currents include the generation of spin torque^3^ for ultrafast magnetization switching and of broadband THz pulses^4,5^ for spectroscopy.

In nanometer-thin *|* stacks consisting of a ferromagnetic-metal layer  and a heavy-metal layer , THz spin transport from  to  can straightforwardly be driven by excitation with a femtosecond laser pulse.^4,6,7^ The resulting spin current *j*_s_(*t*) vs time *t* can be characterized by conversion into a charge current in  and monitoring of the concomitantly emitted THz electromagnetic field. However, the measured THz signal *S* = *S*(*t*) is connected to *j*_s_ by a complex response function, whose determination is not straightforward.^4,6,8^ Even if the temporal evolution of *j*_s_ can be inferred from *S*, it is still convoluted with the dynamics of the force driving the spin current. Consequently, many THz-emission works do not consider the dynamics of *j*_s_(*t*) and rather focus on the peak amplitude, delay and bandwidth of THz signals.^5,9−14^

**Figure 1:**
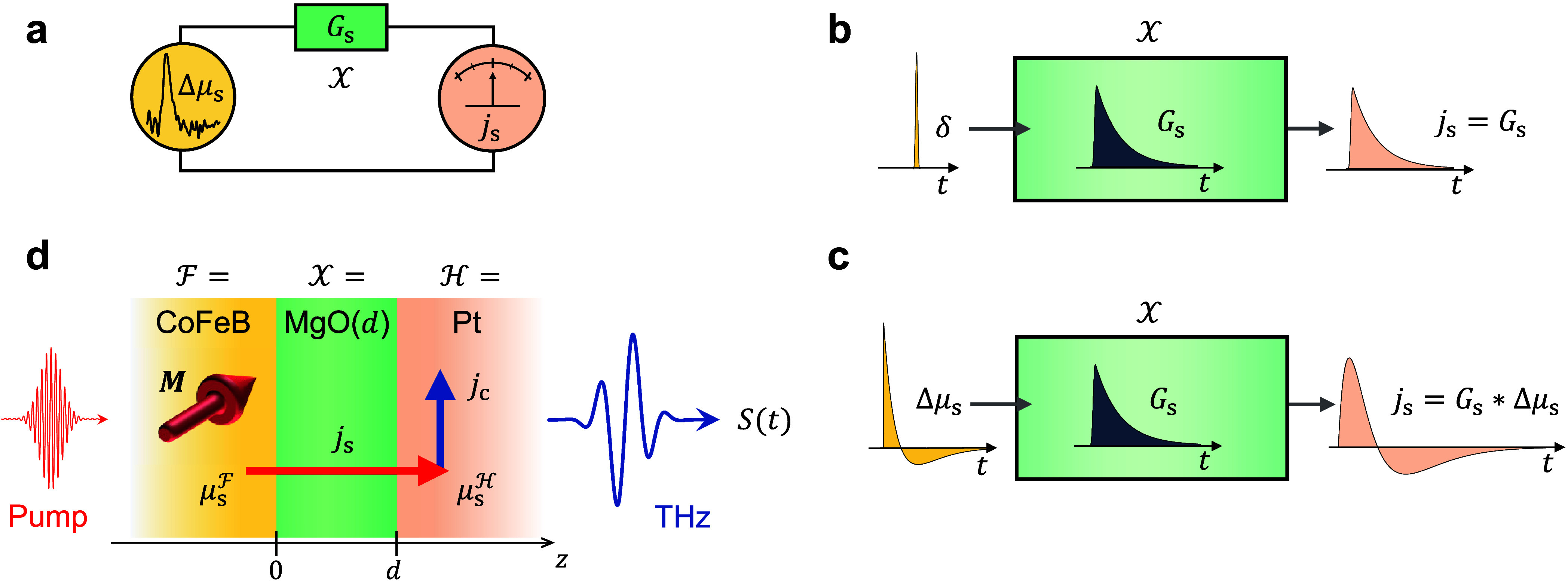
Spin-conductance spectroscopy of a layer  at terahertz frequencies. (a) Schematic of the concept. A transient spin voltage Δμ_s_ vs time *t* is applied across a spin conductor . The resulting spin-current density *j*_s_ is measured by a suitable detector and scales linearly with Δμ_s_ and the spin conductance *G*_s_ of . (b) The dynamics of *G*_s_ = *G*_s_(*t*) with time *t* can simply be understood as the spin current *j*_s_(*t*) that is obtained for an impulsive spin voltage Δμ_s_(*t*) ∝ δ(*t*). (c) For arbitrary spin-voltage dynamics Δμ_s_(*t*), the spin current is given by the convolution *j*_s_ = *G*_s_∗Δμ_s_ (eq 1). By Fourier transformation into the frequency domain, the convolution turns into a simple multiplication *j̃*_s_(ω) = *G̃*_s_(ω)Δμ̃_s_(ω), which can be solved for the spin conductance *G̃*_s_(ω) at each frequency ω/2π. (d) In the experiment, a thin layer  of MgO in a *|**|* thin-film stack is studied, where the ferromagnetic layer  and a heavy-metal layer  serve as spin-current source and detector, respectively. A femtosecond laser pulse induces a transient spin voltage  in  and, thus, spin transport through . The spin current *j*_s_ arriving in  is converted into an in-plane charge current *j*_c_(*t*) ∝ *j*_s_(*t*) and detected by sampling the THz pulse that *j*_c_ emits. By using the measured THz signal *S*(*t*) and eq 3, we determine *G*_s_ for various  thicknesses *d*.

To characterize THz spin transport independently of the shape of driving force and setup transfer function, we borrow concepts from sub-THz spintronics. Here, an important quantity is the spin conductance *G*_s_ = *j*_s_/Δμ_s_ which quantifies how much spin-current density *j*_s_ is obtained when a spin-voltage drop Δμ_s_ is applied across a conductor  (Figure 1a-c). We focus on longitudinal spin transport, which can be described by populations of spin-up and spin-down electron states. At frequencies up to 1 GHz, spin-transport measurements typically rely on electrical contacts.^15−17^ At THz frequencies, however, measurement procedures of *G*_s_ still need to be developed.

In this work, we introduce an approach to measure the spin conductance of a thin film  between a ferromagnetic metal layer  and a heavy-metal layer  (Figure 1d). By using THz-emission spectroscopy,^18^ we obtain the complex-valued spin conductance *G̃*_s_(ω) at frequencies ω/2π = 0.5-12 THz. In the time domain, *G*_s_(*t*) vs time *t* can easily be understood as the spin current that would be obtained for a δ(*t*)-like spin-voltage pulse. Our procedure is successfully demonstrated for a tunnel barrier  made of MgO and reveals dynamic signatures of coherent and incoherent spin tunneling. We expect that THz spin-conductance spectroscopy will provide important insights into ultrafast spin transport in a wide range of materials.

## THz Spin-Conductance Spectroscopy

The general idea of spin-conductance spectroscopy is shown in Figure 1a. A spin voltage Δμ_s_ across spin conductor  drives a spin current *j*_s_ through . Importantly, the response of  is often linear and can, therefore, be fully quantified by applying an impulsive spin voltage Δμ_s_(*t*) = δ(*t*) (Figure 1b). The resulting spin current *j*_s_(*t*) = *G*_s_(*t*) is known as the spin conductance of  in the time domain. It is an example of an impulse-response function, which is a very intuitive concept. For example, any response *G*_s_(*t* > 0) after the excitation at *t* = 0 points to memory effects like the storage and release of spins by  (Figure 1b).

In a typical experiment, however, the spin-voltage dynamics Δμ_s_(*t*) is not impulsive (Figure 1c). In this general case, the resulting spin current is given by the convolution
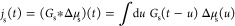
1of *G*_s_ and Δμ_s_ in the time domain. Eq 1 turns into the simple multiplication
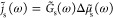
2in the frequency domain, where the tilde denotes Fourier transformation. Therefore, even for a nonimpulsive spin voltage, eq 2 allows us to obtain the spin conductance *G̃*_s_(ω) over the frequency bandwidth of Δμ̃_s_.

To push this concept to THz frequencies, we employ a  stack (Figure 1d). The metallic ferromagnetic layer  (here CoFeB) serves as spin-current source,  is the layer under investigation, and the heavy-metal layer  (here Pt) acts as detector. First, a femtosecond laser pulse instantaneously heats the electrons in . The resulting excess of spin density in , also known as generalized spin voltage^7,19^ of , drives a spin current through . Second, the spin current *j*_s_(*t*) arriving in  is converted into an in-plane charge current with density *j*_c_(*t*) ∝ *j*_s_(*t*) by the inverse spin Hall effect (ISHE). Third, *j*_c_(*t*) gives rise to the emission of a measurable ultrashort THz electromagnetic pulse that is probed by electro-optic sampling, resulting in a signal waveform *S*(*t*) (Figure 1d).^4,6^

To infer *G*_s_ from the signals *S*, we take the following steps, as rationalized in Supporting Notes 1-3. First, a possible spin voltage  in  plays a minor role, that is, . Second, to determine , we conduct a reference measurement on a sample without interlayer , where  with known *G̃*^ref^(ω) (eq 2). Third, *S̃* and *j̃*_s_ are related by a response function that greatly cancels when the reference measurement is considered.^8^ Consequently, the spin conductance *G̃*_s_ of , normalized to the reference conductance *G̃*^ref^(ω), is fully and simply determined by the observables *S̃* and *S̃*^ref^ through
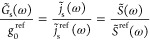
3In the last step of eq 3, we took advantage of the facts that the measured optical absorptance and THz impedance of all the samples in our experiment are the same and, thus, cancel, and that the spin conductance *G̃*^ref^(ω) = *g*_0_^ref^ of the | interface is constant over the frequency interval considered here (see Supporting Notes 1-3).

## Experimental Details

To put the scheme of Figure 1d to the test, we apply it to the nonmagnetic insulator  as a function of its thickness *d*. MgO has been extensively used as a tunnel barrier in magnetic tunnel junctions that provide large tunneling magnetoresistance for applications in nonvolatile memory devices.^20−23^ The samples are
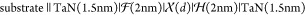
stacks with , various MgO thicknesses of 0 ≤ *d* ≤ 15 Å and . The sample with *d* = 0 is the reference sample without . All layers are grown on MgO substrates by DC magnetron sputtering at a base pressure of 4 × 10^–3^ mbar, except the MgO layer, which is grown in the same vacuum chamber by radio frequency sputtering using an off-axis gun tilted by 90° from the substrate plane.^11^ Atomic-force microscopy shows a roughness of <2 Å for all layers.^11^

In the THz-emission experiments (Figure 1d), the magnetization ***M*** of  is saturated by an external magnetic field of about 10 mT parallel to the sample surface and perpendicular to the surface of the optical table. The sample is excited by a train of linearly polarized ultrashort laser pulses (central wavelength 790 nm, nominal pulse duration 10 fs, pulse energy 2 nJ, repetition rate 80 MHz) from a Ti:sapphire laser oscillator. The beam is normally incident onto the sample from the substrate side, traverses the substrate and arrives at the *|**|* stack with a focus size of 30 μm full width of half-maximum (FWHM) of intensity. The THz pulse that is emitted from the stack toward the air side is collimated and refocused by two 90°-off-axis parabolic mirrors into an electro-optic GaP(110) crystal (thickness 250 μm). Here, the THz electric field is sampled by linearly polarized probe pulses (0.6 nJ) from the same laser. This procedure yields the THz signal *S*(*t*,***M***), i.e., the THz-field-induced probe ellipticity, as a function of the delay *t* between probe and THz pulse.^7^

As we are primarily interested in the charge current *j*_c_(*t*) ∝ *j*_s_(*t*) arising from the spin current from  and the inverse spin Hall effect in , we detect the THz electric-field component perpendicular to the  magnetization ***M*** and only consider signals odd in ***M***, i.e., *S*(*t*) = [*S*(*t*,+***M***) – *S*(*t*, –***M***)]/2. Signal components even in ***M*** are minor. The origin of the *t* axis may vary from sample to sample due, e.g., to variations of substrate thickness. Such shifts are typically <10 fs here and irrelevant, because we do not focus on possible *d*-dependent signal delays.^24^

## THz Signals

**Figure 2:**
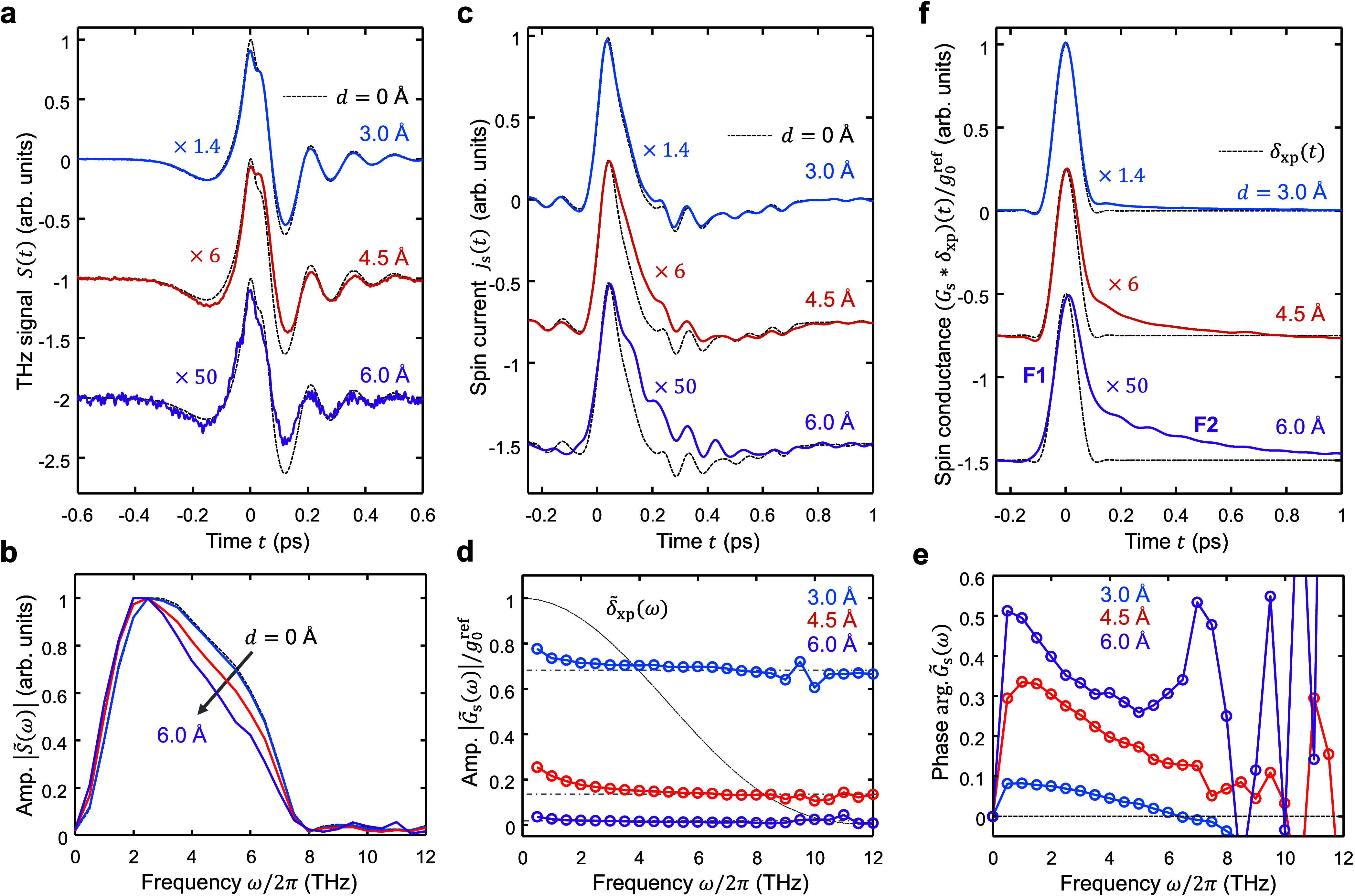
From THz signals to spin currents to spin conductance for CoFeB(2 nm)|MgO(*d*)|Pt(2 nm). (a) Time-domain electro-optic THz signals *S*(*t*) odd in the CoFeB magnetization ***M*** for various MgO thicknesses of *d* = 0, 3.0, 4.5, and 6.0 Å. The signal for *d* = 0 is the reference signal *S*^ref^ = *S|*_*d*=0_ (dashed lines). (b) Fourier amplitude spectra of the signals shown in panel (a). Maxima are normalized to unity. (c) Spin current *j*_s_(*t*) extracted from the THz signals shown in panel (a). The dashed lines correspond to *j*_s_(*t*)|_*d*=0_ ∝  (d) Amplitude of the frequency-domain spin conductance |*G̃*_s_(ω)/*g*_0_^ref^| vs frequency ω/2π, where *g*_0_^ref^ = *G̃*_s_(ω)|_*d*=0_ is found to be ω-independent. The function δ̃_xp_(ω) (dashed black line) indicates the finite frequency bandwidth of our setup. (e) Spectral phase arg *G̃*_s_(ω). (f) Time-domain THz spin conductance of MgO barriers with various thicknesses *d* (thin solid lines). Due to the nonzero time resolution, the δ-function is broadened to a fictitious impulsive spin voltage δ_xp_(*t*) (dashed lines), which equals the inversely Fourier-transformed δ̃_xp_(ω) in panel (e). Accordingly, the extracted conductivity curves are also broadened and display (*G*_s_∗δ_xp_)(*t*). For clarity, curves in panels (a, c, f) are offset vertically.

Figures 2a and S1 show THz-emission signals *S*(*t*) odd in ***M*** from CoFeB|MgO|Pt stacks for various MgO thicknesses *d*. The signal for *d* = 0 (Figure 2a) is dominated by ultrafast spin transport from CoFeB into Pt, where *G̃*_s_(ω)|_*d*=0_ = *g*_0_^ref^ = const_ω_ (Supporting Note 4 and ref (7)).

When *d* is increased from 0 to 6.0 Å, the signal amplitude decreases by a factor of 60 (Figure 2a). One can show that, in this range, the THz signal is dominated by spin transport from  through MgO into  and the ISHE in , consistent with the signal origin for *d* = 0. For *d* > 6.0 Å, the signals are increasingly superimposed by THz emission from layer  and, therefore, not considered further (see Supporting Note 4).

Interestingly, with increasing *d*, the signal amplitude drops, and the initially sharp temporal features become wider (Figure 2a). This trend suggests the emergence of slower components. It is confirmed by the normalized Fourier spectra (Figure 2b), whose width decreases with increasing *d* and whose maximum shifts to lower frequencies.

## Spin Currents

By applying an inversion procedure to the signals in Figure 2a (see Supporting Note 1), we extract the spin-current dynamics *j*_s_(t) shown in Figure 2c. The spin current for the | reference stack (*d* = 0, dashed line) has the same dynamics as the spin voltage of , i.e.,^7^. At arrival of the pump,  rises instantaneously, subsequently decreases with a rate given by the electron-spin relaxation time of CoFeB (≈100 fs), turns slightly negative and finally decays with a rate given by electron-phonon equilibration (≈400 fs).^7,25^

When the MgO thickness *d* increases from 0 to 6.0 Å, the amplitude of the spin current decreases, and its relaxation dynamics slows down (Figure 2c). For example, the *j*_s_(*t*) for *d* = 6.0 Å is 50 times smaller and relaxes substantially more slowly than the spin current measured in the reference sample (*d* = 0, dashed line).

## Frequency-Domain Spin Conductance

Note that the spin currents  still contain the relatively complicated dynamics  of the spin voltage (Figure 2c). To gain more intrinsic information on the dynamics of spin transport through MgO, we apply eq 3 to the raw signals (Figure 2a) in the frequency domain (Figure 2b) and determine the normalized spin conductance *G̃*_s_(ω)/*g*_0_^ref^ as a function of frequency ω/2π = 0.5-12 THz. We emphasize that this step does not require any knowledge of spin currents *j*_s_(*t*) nor transfer functions.

The modulus |*G̃*_s_(ω)/*g*_0_^ref^| (Figure 2d) consists of a frequency-independent component (dashed-dotted lines) and an additional contribution below 3 THz. As expected from Figure 2a, we observe a drastic overall amplitude reduction with increasing *d*. At the same time, spectral weight is shifted to frequencies below 3 THz. The spectral phase arg *G̃*_s_(ω) (Figure 2e) of the spin conductance and its slope vs ω increase with *d*, indicating an increasingly noninstantaneous response.

## Time-Domain Spin Conductance

To obtain the time-domain spin conductance *G*_s_(*t*), one needs to inversely Fourier-transform *G̃*_s_(ω). This task is not trivial because the Fourier amplitudes *S̃*(ω) of the THz signal are relatively small for frequencies ω/2π > 8 THz (Figure 2b). They are, thus, prone to unwanted signal contributions like noise, which lead to the strong random oscillations of the spectral phase arg *G̃*_s_(ω) for ω/2π > 8 THz (Figure 2e).

To suppress these spectral components, we multiply *G̃*_s_ with a suitable window function δ̃_xp_. The product *G̃*_s_δ̃_xp_ in the frequency domain results in the convolution *G*_s_*∗δ*_xp_ of *G*_s_ with δ_xp_ in the time domain. To achieve a δ_xp_(*t*) that is a single unipolar peak with a duration as short as possible, we use a Norton-Beer function^26,27^ for δ̃_xp_(ω), which approaches zero at 12 THz (dashed line in Figure 2d). As a consequence, *G*_s_∗δ_xp_ is a low-pass-filtered *G*_s_, where possible sharp features are smeared over the width of δ_xp_ (dashed lines in Figure 2f). In other words, the 90 fs FWHM of δ_xp_(*t*) defines the time resolution of the extracted *G*_s_(*t*).

The *G*_s_(*t*) traces (Figure 2f) consist of an initial instantaneous feature (F1) with a shape similar to δ_xp_(*t*) and a subsequently tail-like feature (F2), whose relative weight increases with *d*. Component (F2) is fully consistent with the slower relaxation of the spin current *j*_s_(*t*) relative to the spin voltage  (Figure 2c). However, the *G*_s_(*t*) traces much more clearly reveal that two features (F1) and (F2) generate the response of the MgO barrier. The reason is that the driving force of *G*_s_, the fictitious impulsive spin voltage δ_xp_ ≈ δ (Figures 1b and 2f), has a much simpler structure than the experimental spin voltage  that induces the measured spin current *j*_s_ (Figures 1c and 2c).

Importantly, based on THz emission from samples tilted by ±45°, we estimate that possible pump-induced THz charge currents along the *z* axis (Figure 1d) are at least 2 orders of magnitude smaller than the in-plane currents *j*_c_. Therefore, our extracted spin conductance *G*_s_(*t*) across  is measured under the boundary condition of negligible out-of-plane charge current.

## Interpretation

**Figure 3:**
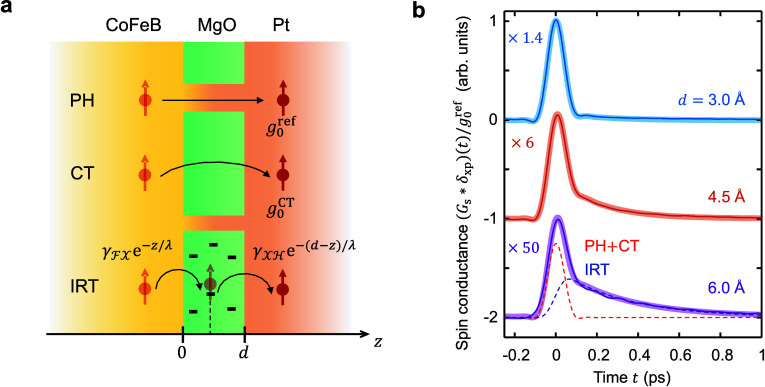
Interpretation of the THz spin conductances of MgO. (a) Spin-transport channels through MgO: flow through conducting pinholes (PH), coherent tunneling (CT), and incoherent resonant tunneling (IRT). For IRT,  is the probability of an electron tunneling from  to a defect at position *z* in , whereas  is the tunneling probability from *z* to . (b) Measured THz spin conductance (solid thin lines, from Figure 2f) along with fits (thick solid lines) based on eq 5. For *d* = 6 Å, the dashed lines show the instantaneous [*Aδ*_xp_(*t*)] and noninstantaneous [*B*Θ(*t*)e^–*t*/τ^] contribution, which is, respectively, ascribed to channels PH+CT (red dashed line) and IRT (blue dashed line).

To interpret the dynamics of *G*_s_(*t*) (Figure 2f), we briefly review the relevant known static properties of MgO thin films. For *d* < 10 Å, structural imperfections are reported, in particular oxygen vacancies^28−33^ and pinholes^34,35^ connecting  and . Accordingly, we consider three possible contributions to the total spin current through MgO in CoFeB|MgO|Pt stacks (Figure 3a): spin transport through Pt- or CoFeB-filled pinholes (PH) in the MgO film,^35,36^ coherent off-resonant electron tunneling (CT) through the MgO barrier^37−39^ and incoherent resonant tunneling (IRT) through intermediate defect states in the vicinity of the Fermi energy of the CoFeB and Pt layer.^30,33,40−42^ As the three channels PH, CT and IRT add up independently (Figure 3a), the spin conductance is the sum
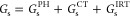
4While the PH and CT processes are instantaneous^7,43^ on the time scale of our experimental resolution of 90 fs (dashed line in Figure 2f), the IRT mechanism may require more time to proceed.

To find analytic expressions for *G*_s_^PH^, *G*_s_^CT^ and *G*_s_^IRT^, we discuss each mechanism in more detail. Regarding *G*_s_^PH^, one expects that, for *d* < 6 Å, MgO grows as islands^34^ or exhibits pinholes.^35^ The pinholes are filled with Pt of the subsequently grown Pt layer and, thus, provide a conductive channel between the CoFeB and Pt layer (Figure 3a). As *G*_s_^PH^ is proportional to the in-plane areal pinhole fraction 0 ≤ *f*^PH^ ≤ 1 and instantaneous, we model it by *G*_s_^PH^(*t*) = *f*^PH^*g*_0_^ref^δ(*t*). Likewise, CT (Figure 3a) through the entire MgO barrier is instantaneous on the scale of our time resolution.^43^ Therefore, *G*_s_^CT^(*t*) = (1–*f*^PH^)*g*_0_^CT^δ(*t*), where *g*_0_^CT^ is the amplitude of the impulsive spin current in the absence of pinholes.

Regarding IRT, oxygen vacancies are known to provide localized electronic states within the MgO band gap and, thus, open up a resonant transport channel.^28,31,32^ In the simplest IRT model, an electron tunnels from  into a MgO vacancy and, subsequently, into  (Figure 3a), similarly to resonant tunneling in quantum wells.^44−47^ One can quantify the resonant tunneling as *G*_s_^IRT^(*t*) = (1–*f*^PH^)*g*^IRT^(*t*), where *g*^IRT^(*t*) is the IRT-related spin conductance of an MgO barrier without pinholes. Based on Figure 3a, we model *g*^IRT^(*t*) by a single-sided exponential decay g_0_^IRT^Θ(*t*)e^–*t*/τ^, where Θ(*t*) is the Heaviside step function and τ can be considered as the characteristic time of IRT.

With these specifications, eq 4 turns into
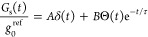
5where
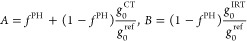
6We fit the convolution *G*_s_∗δ_xp_ of *G*_s_ (eq 5) with our time resolution δ_xp_ to the measured time-domain spin conductance (Figure 2f). Here, the free parameters are τ, *A*, *B* and a possible deviation *t*_0_ from time zero, i.e., *t* → *t*–*t*_0_ in eq 5, due to substrate-thickness variations.^9^ We find that |*t*_0_| is typically smaller than 10 fs (see Supporting Note 5).

**Figure 4:**
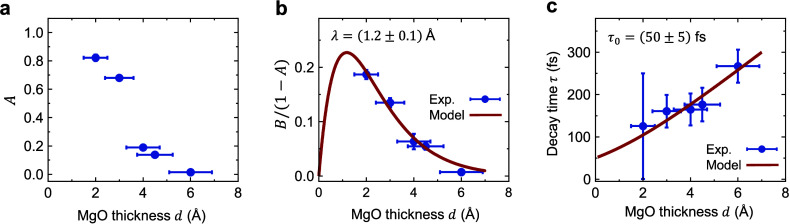
Parameters of the MgO spin conductance *G*_s_(*t*). (a) Amplitude *A* ≈ *f*^PH^ of the instantaneous, i.e., δ_xp_-like component of *G*_s_ vs MgO thickness *d* (blue circles). (b) Amplitude *B*/(1–*A*) ≈ *g*_0_^IRT^/*g*_0_^ref^ of the longer-lived *G*_s_ component vs MgO thickness (blue circles). The red solid line is a fit based on eq 7 with λ = (1.2 ± 0.1) Å. (c) Decay time τ of the IRT current component vs MgO thickness (blue circles). The red solid line is a fit based on eq 7 with τ_0_ = (50 ± 5) fs.

The model fits (Figure 3b) describe our data excellently. The resulting amplitude *A* of the δ-like contribution (Figure 4a) decreases monotonically with MgO thickness *d*, where one can show that *A* ≈ *f*^PH^ (see Supporting Note 6). This assignment and Figure 4a are consistent with previous work^35^ in which *f*^PH^ was found to decrease with increasing MgO thickness *d* on a scale of 2 Å. According to calculations,^11^ MgO layers close to the CoFeB and Pt interface are slightly metallic. They have a total thickness of about 4 Å and can be understood to have a large PH fraction. This effect may explain the pronounced drop of *A* when *d* is increased from 3.0 to4.0 Å (Figure 4a).

Using *A* ≈ *f*^PH^, we can determine *g*_0_^IRT^/*g*_0_^ref^ ≈ *B*/(1 – *A*), i.e., the peak amplitude of the IRT contribution (eq 5), which is found to decrease strongly with increasing *d* (Figure 4b and Supporting Note 6). Remarkably, the characteristic IRT time τ is found to grow with *d* (Figure 4c), consistent with the increase of the slope of arg *G̃*_s_(ω) vs ω (Figure 2e). As a side effect, the shape of *G*_s_(*t*) attains an asymmetry with increasing *d* that, after convolution with δ_xp_, causes a shift of the peak of *G*_s_∗δ_xp_.

## Model of Dynamic IRT

Qualitatively, we suggest the following dynamic scenario for the IRT conductance *g*^IRT^(*t*) ∝ Θ(*t*)e^–*t/τ*^. At time *t* = 0, a δ(*t*)-like spin voltage in CoFeB drives instantaneous tunneling of spin-polarized electrons from CoFeB to MgO defect states (Figure 3a). Simultaneously, spin-unpolarized electrons are transferred from Pt to CoFeB to maintain local charge neutrality.

The subsequent tunneling events from occupied defect states to CoFeB or Pt are stochastic. Their rate *∂N*^σ^/*∂t* is proportional to the number *N*^σ^ of spins σ = ↑ or ↓ occupying the defect state. Accordingly, we obtain (i) a simple temporal exponential decay of *N*^σ^ and, thus, the σ-current from defects to Pt. (ii) The characteristic time of this process increases with increasing *d* because the tunneling from defects to Pt becomes less likely as *d* and, thus, the average distance of defect and Pt grow. As two tunneling events over distance *d* are involved, (iii) the spin current from CoFeB to Pt decreases with increasing *d*. The implications (i), (ii), (iii) of our model are fully consistent with the experimental results of Figures 3b, 4c and 4b, respectively.

More quantitatively, a rate-equation treatment and the assumption of vanishing out-of-plane charge current (see Supporting Note 7) reproduce the relationship *g*^IRT^(*t*) = *g*_0_^IRT^Θ(*t*)e^–*t*/τ^ with
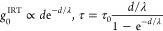
7Here, τ_0_ quantifies the spin lifetime in a defect state for an infinitely thin MgO layer with *d* ≪ λ, where λ is the spin decay length in a MgO barrier. Eq 7 provides good fits to the data of Figure 4b, 4c and yields τ_0_ = (50 ± 5) fs and λ = (1.2 ± 0.1) Å, in agreement with previous measurements.^20,31,38,48,49^

## Discussion

The extracted λ value is up to 30% smaller than previously reported relaxation lengths^20,31,49^ for at least two reasons. First, when *d* rises, the defect density in MgO is not constant but rather drops, resulting in an underestimation of λ. This effect may depend strongly on the MgO crystallinity^20,50,51^ and could be addressed in future experiments. Second, the tunneling probability is spin-dependent with decay lengths^38^ λ^↓^ < λ^↑^. However, because no net charge is transported through MgO in our experiment, we measure the smaller λ^↓^, i.e., λ ≈ λ^↓^ (see Supporting Note 7).

The spin-current relaxation time τ in defect states increases with the thickness of MgO, reaching as much as 270 fs for *d* = 6.0 Å. It is important to note that *g*_0_^IRT^, τ_0_ and, thus, τ depend on the electron transparency of the / and / interfaces (see Supporting Note 7). Therefore, the magnitude and dynamics of the spin conductance *G*_s_(*t*) across a layer  depend not only on the bulk properties of , but also on its interfaces. However, the main characteristics of the IRT-based spin conductance of MgO remain unchanged: the spin decay length λ and the increase of the relaxation time τ with the MgO thickness. To gain more insight into the impact of the  and / interfaces on *g*^IRT^(*t*), future studies may measure the THz spin conductance of MgO barriers as a function of the  and  material.

We emphasize that determination of the THz spin conductance (Figure 2f) relies on the measurement of just two THz emission signals (eq 3), without having to know any instrument response functions, in contrast to extraction of the spin current *j*_s_ (Figure 2c). This approach can be extended to, in principle, any  material other than MgO, if the following three assumptions are fulfilled: (A1) The signal exclusively arises from the spin current *j*_s_ arriving in . (A2) The *j*_s_ solely originates from the spin voltage  and is dominated by . (A3) The presence of the layers  and  in the stack || does not change the dynamics of .

For our CoFeB|MgO(*d*)|Pt system, (A1) is fulfilled for the reference sample (*d* = 0), but also for *d* ≤ 6 Å because the ISHE of Pt dominates spin-to-charge conversion of the metal stack (Supporting Note 4). (A2) is valid for the reference sample^7^ and, thus, for *d* ≠ 0, too (Supporting Note 3). (A3) is fulfilled for the reference sample (Supporting Note 3) and, thus, for *d* > 0 also. If layer  changes the pump absorptance and THz conductivity of the sample stack, their effect needs to be accounted for in eq 3 (Supporting Note 2). We again emphasize that the spin conductance determined with our approach (Figure 1d) refers to a situation with negligible net charge transport.

In conclusion, we demonstrate a new method, THz spin-conductance spectroscopy, to study ultrafast spin transport across a layer . We apply it to the example of MgO barriers and find an ultrafast signature of IRT: a spin-current relaxation tail whose decay time increases with the barrier thickness because the tunneling probability decreases. Our method inherits all benefits of THz-emission spectroscopy,^18,52−56^ in particular contact-free operation with large sample throughput. Future work may push the time resolution down to the 10 fs scale of electron-momentum relaxation.^57,58^ In this way, THz spin-conductance spectroscopy will provide significant means to separate elementary transport processes from ballistic to diffusive in a wide range of spintronic nanostructures made of simple metals^59^ or complex materials like antiferromagnets.^60−62^

## Supporting Information Available

Supporting Figure S1: THz emission signals for CoFeB(3 nm)|MgO(*d*)|Pt(3 nm) with 0 ≤ *d* ≤ 15 Å. Supporting Figure S2: THz emission from | vs  samples. Supporting Note 1: Extraction of spin current from THz signals. Supporting Note 2: THz spin conductance of layer . Supporting Note 3: Spin conductance of the CoFeB/Pt interface and Pt spin voltage. Supporting Note 4: Prevalence of spin transport from CoFeB to Pt for *d* ≤ 6 Å. Supporting Note 5: Fitting by eq 5. Supporting Note 6: Analysis of *A* and eq 6. Supporting Note 7: Rate-equation model of IRT. (PDF)
