# Supplementary material for: Terahertz Spin-Conductance Spectroscopy: Probing Coherent and Incoherent Ultrafast Spin Tunneling
Source: Nano Lett. 2024 Jun 21;24(26):7852–60. doi: 10.1021/acs.nanolett.4c00498 (PMC11229073; doi:10.1021/acs.nanolett.4c00498)
Supplement: Supplementary file 1 — nl4c00498_si_001.pdf [file nl4c00498_si_001.pdf]

Supporting Information for

Terahertz spin-conductance spectroscopy: probing coherent and incoherent ultrafast spin tunneling

Reza Rouzegar[1,2*], Mohamed Amine Wahada[3,4], Alexander L. Chekhov[1,2], Wolfgang Hoppe[4], Genaro Bierhance[1,2], Jiří Jechumtál[5], Lukáš Nádvorník[5], Martin Wolf[2], Tom S. Seifert[1], Stuart S. P. Parkin[3], Georg Woltersdorf[4], Piet W. Brouwer[1], Tobias Kampfrath[1,2]

1. Department of Physics, Freie Universität Berlin, 14195 Berlin, Germany
2. Department of Physical Chemistry, Fritz Haber Institute of the Max Planck Society, 14195 Berlin, Germany
3. Max Planck Institute for Microstructure Physics, Weinberg 2, 06120 Halle, Germany
4. Institut für Physik, Martin-Luther-Universität Halle, 06120 Halle, Germany
5. Faculty of Mathematics and Physics, Charles University, Ke Karlovu 3, 121 16 Prague, Czech Republic

m.rouzegar@fu-berlin.de

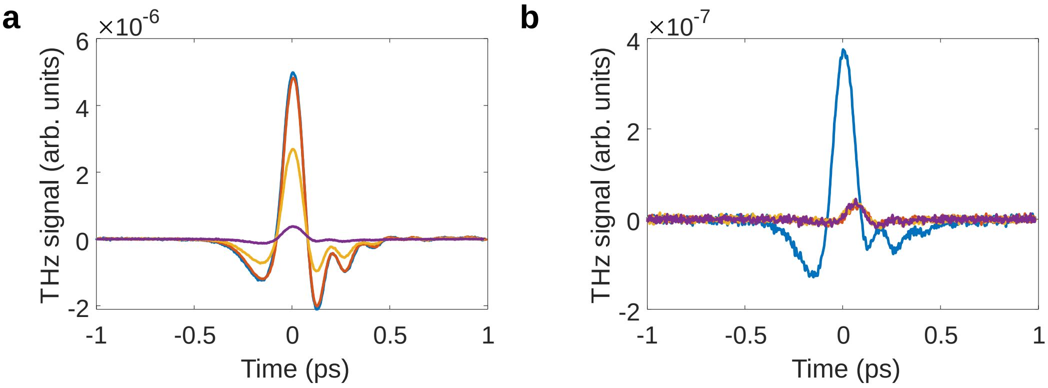

Fig. S1. THz emission signal $S(t)$ from CoFeB(3 nm)|MgO(d)|Pt(3 nm) stacks odd in the CoFeB magnetization. **(a)** Signals for MgO thicknesses of 0 Å $\leq d \leq$ 5 Å and of **(b)** 5 Å $\leq d \leq$ 15 Å.

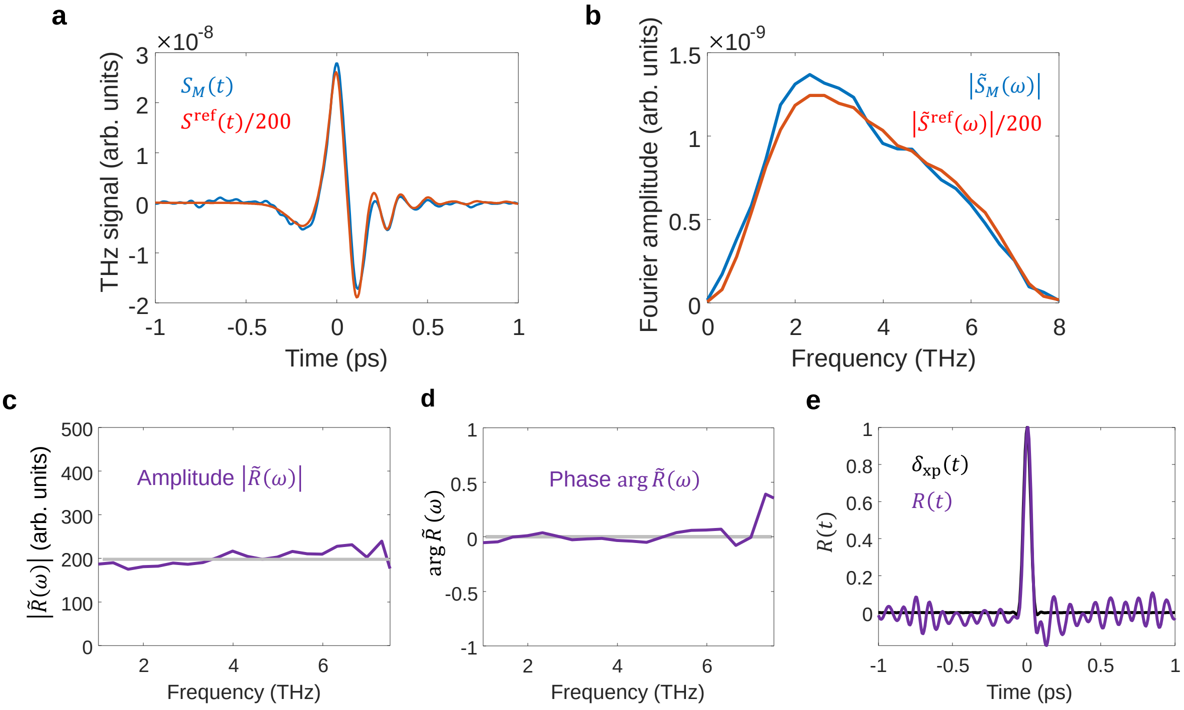

Fig. S2. THz emission from $\mathcal{F}|\mathcal{H}$ vs $\mathcal{F}$ samples. **(a)** THz-emission signal $S_M(t)$ resulting from ultrafast demagnetization of a single layer $\mathcal{F}$ = CoFeB(5 nm), and a signal $S^{\mathrm{ref}}(t)$ resulting from ultrafast spin transport from layer $\mathcal{F}$ to $\mathcal{H}$ in CoFeB(5 nm)|Pt(3 nm). **(b)** Fourier amplitude of THz signals shown in panel (a). **(c)** Fourier amplitude of $\tilde{R}(\omega) = \tilde{S}_M(\omega)/\tilde{S}^{\mathrm{ref}}(\omega)$ and **(d)** spectral phase $\arg \tilde{R}(\omega)$. **(e)** Time-domain version $R(t)$ (blue line). The $\delta_{\mathrm{xp}}(t)$ quantifies our experimental time resolution (black line). Curves are normalized to one.

Supporting Notes

Supporting Note 1. Extraction of spin current from THz signals

The emitted THz electric field for $\mathcal{F}|\mathcal{X}|\mathcal{H}$ sample is given by

$$\tilde{E}(\omega) = e\tilde{Z}(\omega)\tilde{\theta}_{\mathrm{SH}}(\omega)\tilde{\lambda}_{\mathrm{rel}}(\omega)\tilde{j}_{\mathrm{s}}(\omega), \tag{S1}$$

where $-e$ is the electron charge, $\tilde{Z}(\omega)$ is the impedance of the $\mathcal{F}|\mathcal{H}$ reference sample, $\tilde{\theta}_{\mathrm{SH}} = \tilde{j}_{\mathrm{c}}/\tilde{j}_{\mathrm{s}}$ is the spin Hall angle of $\mathcal{H} =$ Pt and $\tilde{\lambda}_{\mathrm{rel}}$ is the spin relaxation length in $\mathcal{H} =$ Pt. The measured THz signal $\tilde{S}(\omega)$ at the detector position is connected to the emitted THz electric field $\tilde{E}(\omega)$ directly behind the sample through

$$\tilde{S}(\omega) = \tilde{H}_{SE}(\omega)\tilde{E}(\omega). \tag{S2}$$

Here, $\tilde{H}_{SE}(\omega)$ is the setup transfer function that captures THz propagation to the detector and the electro-optic sampling process. Note that the case $d = 0$ coincides with the $\mathcal{F}|\mathcal{H}$ reference sample. We determine $\tilde{H}_{SE}(\omega)$ by using a well-understood emitter[1], GaP(110), with a thickness of 50 µm. Eqs. (S1) and (S2) imply that the spin current can be extracted by

$$\tilde{j}_{\mathrm{s}}(\omega) = \frac{\tilde{S}(\omega)}{\tilde{H}_{SE}(\omega)} \frac{1}{e\tilde{Z}(\omega)\tilde{\theta}_{\mathrm{SH}}(\omega)\tilde{\lambda}_{\mathrm{rel}}(\omega)}. \tag{S3}$$

The impedance for $\mathcal{F}|\mathcal{X}|\mathcal{H}$ is measured by THz transmission spectroscopy[2], and we find $\tilde{Z}(\omega) = \tilde{Z}^{\mathrm{ref}}(\omega) = 80\,\Omega$ for all d (see Fig. S4 in Ref. 3). The $\tilde{\theta}_{\mathrm{SH}}$ and $\tilde{\lambda}_{\mathrm{rel}}$ are assumed to be frequency-independent. Typical signals are shown in Figs. 2a and S1, and extracted spin currents are shown in Fig. 2c.

Supporting Note 2. THz spin conductance of layer $\mathcal{X}$

As seen in Supporting Note S1, the extraction of the setup transfer function $\tilde{H}_{SE}$ and, thus, the spin current from the THz-emission signal is not straightforward. However, the determination of the THz spin conductance $\mathcal{X}$ does not require knowledge of $\tilde{H}_{SE}$ and is, therefore, much more easily to implement.

The spin current $\tilde{j}_{\mathrm{s}}(\omega)$ arriving in layer $\mathcal{H}$ in the $\mathcal{F}|\mathcal{X}|\mathcal{H}$ sample is given by

$$\tilde{j}_{\mathrm{s}}(\omega) = \tilde{G}_{\mathrm{s}}(\omega)\Delta\tilde{\mu}_{\mathrm{s}}(\omega). \tag{S4}$$

Here, $\Delta\mu_{\mathrm{s}} = \mu_{\mathrm{s}}^{\mathcal{F}} - \mu_{\mathrm{s}}^{\mathcal{H}}$ is the difference of the transient spin voltage $\mu_{\mathrm{s}}^{\mathcal{F}}$ and $\mu_{\mathrm{s}}^{\mathcal{H}}$ between layer $\mathcal{F}$ and $\mathcal{H}$, respectively, and $\tilde{G}_{\mathrm{s}}(\omega)$ is the spin conductance of layer $\mathcal{X}$. The connection of $\tilde{j}_{\mathrm{s}}(\omega)$ to the actually measured signal $\tilde{S}(\omega)$ is given by Eqs. (S1) and (S2).

To access the spin conductance $\tilde{G}_{\mathrm{s}}(\omega)$ without knowledge of the setup response function $\tilde{H}_{SE}(\omega)$ [Eq. (S2)], we consider $\mathcal{F}|\mathcal{H}$ as suitable reference sample, which is identical to all the $\mathcal{F}|\mathcal{X}|\mathcal{H}$ samples apart from a lacking layer $\mathcal{X}$ ($d = 0$). If the $\mathcal{F}|\mathcal{X}|\mathcal{H}$ sample and the $\mathcal{F}|\mathcal{H}$ reference sample are placed at the same position on the optical axis, the two resulting THz electric fields directly behind the metal stack are at the same position on the optical axis and have the same relevant lateral spatial distribution[2]. Therefore, the connection between signal and field is described by the same instrument response function $\tilde{H}_{SE}(\omega)$, even though the pump-beam diameter of about 30 µm is comparable or even smaller than the relevant THz wavelengths[2].

Likewise, $\tilde{\theta}_{\mathrm{SH}}(\omega)$ and $\tilde{\lambda}_{\mathrm{rel}}(\omega)$ are the same for both $\mathcal{F}|\mathcal{H}$ and $\mathcal{F}|\mathcal{X}|\mathcal{H}$. As shown previously[4] and in Supporting Note S3, the dynamics $\Delta\tilde{\mu}_{\mathrm{s}}(\omega)$ of the spin voltage is strongly dominated by the spin-voltage dynamics in $\mathcal{F}$, i.e.,

$$\Delta\mu_{\mathrm{s}} = \mu_{\mathrm{s}}^{\mathcal{F}} - \mu_{\mathrm{s}}^{\mathcal{H}} = \mu_{\mathrm{s}}^{\mathcal{F}}. \tag{S5}$$

Consequently, by applying Eqs. (S2)-(S4) to both the sample signal $\tilde{S}(\omega)$ and the reference signal $\tilde{S}^{\mathrm{ref}}(\omega)$ and taking their ratio, we obtain

$$\frac{\tilde{S}(\omega)}{\tilde{S}^{\mathrm{ref}}(\omega)} = \frac{\tilde{G}_{\mathrm{s}}(\omega)}{\tilde{G}_{\mathrm{s}}^{\mathrm{ref}}(\omega)} \frac{\tilde{Z}(\omega)}{\tilde{Z}^{\mathrm{ref}}(\omega)}. \tag{S6}$$

Here, $\tilde{G}_s^{\mathrm{ref}}(\omega) = \tilde{G}_s(\omega)\big|_{d=0}$ and $\tilde{Z}^{\mathrm{ref}}(\omega) = \tilde{Z}(\omega)\big|_{d=0}$ is, respectively, the spin conductance and impedance of the reference sample.

Finally, as shown in Supporting Note S3, the (interface) spin conductance $\tilde{G}_s^{\mathrm{ref}}(\omega) = g_0^{\mathrm{ref}}$ of the $\mathcal{F}|\mathcal{H}$ reference sample is frequency-independent, and Eq. (S6) turns into

$$\frac{\tilde{G}_s(\omega)}{g_0^{\mathrm{ref}}} = \frac{\tilde{S}(\omega)}{\tilde{S}^{\mathrm{ref}}(\omega)} \frac{\tilde{Z}^{\mathrm{ref}}(\omega)}{\tilde{Z}(\omega)}. \tag{S7}$$

To summarize, the unknown $\tilde{\theta}_{\mathrm{SH}}(\omega)$, $\tilde{\lambda}_{\mathrm{rel}}(\omega)$ and $\tilde{H}_{SE}(\omega)$ all cancel in Eq. (S7), and one can measure the THz spin conductance of a layer $\mathcal{X}$ without knowledge of the setup transfer function and the difficult-to-measure $\tilde{\theta}_{\mathrm{SH}}(\omega)$ and $\tilde{\lambda}_{\mathrm{rel}}(\omega)$.

The impedance for $\mathcal{F}|\mathcal{X}|\mathcal{H}$ is determined by THz transmission spectroscopy. Because we find $\tilde{Z}(\omega) = \tilde{Z}^{\mathrm{ref}}(\omega) = 80\ \Omega$ for all d (see Fig. S4 in Ref. 3), Eq. (S7) simplifies to Eq. (3) of the main text.

Supporting Note 3. Spin conductance of the CoFeB/Pt interface and Pt spin voltage

To determine the conductance of the CoFeB/Pt interface of the CoFeB|Pt reference stack, we directly interrogate the spin-voltage dynamics $\mu_s^{\mathcal{F}}(t)$ of $\mathcal{F}$ by measuring the rate of change $\partial_t M = \partial M/\partial t$ of the magnetization of a single film $\mathcal{F}$ = CoFeB, which fulfills[4] $\partial_t M(t) \propto \mu_s^{\mathcal{F}}(t)$. The emitted THz field symmetric with respect to sample turning by 180° about the magnetization vector is dominated by magnetic-dipole radiation due to[4] $\partial_t M$. In the frequency domain, the THz field amplitude directly behind the sample is

$$\tilde{E}_M(\omega) = -\frac{\tilde{Z}_{\mathcal{F}}(\omega)\tilde{n}(\omega)d_{\mathcal{F}}}{c}\,\mathrm{i}\omega\tilde{M}(\omega), \tag{S8}$$

where $\tilde{n}(\omega)$ is the refractive index of the substrate, $d_{\mathcal{F}}$ is the $\mathcal{F}$ thickness, $\tilde{Z}_{\mathcal{F}}(\omega)$ is the impedance of the $\mathcal{F}$ sample and c is the speed of light[4]. On the other hand, the rate of magnetization change $\mathrm{i}\omega\tilde{M}(\omega)$ is given by[4]

$$\mathrm{i}\omega\tilde{M}(\omega) = 2g_{\mathrm{sf}}\,\tilde{\mu}_s^{\mathcal{F}}(\omega), \tag{S9}$$

where the coefficient g_{sf} quantifies the spin-flip strength of $\mathcal{F}$.

Note that $\tilde{S}_M(\omega)$ and $\tilde{S}^{\mathrm{ref}}(\omega)$ are measured under identical experimental conditions. Therefore, the measured THz signal $\tilde{S}_M(\omega)$ is given by $\tilde{S}_M(\omega) = \tilde{H}_{SE}(\omega)\tilde{E}_M(\omega)$ with an identical setup transfer function $\tilde{H}_{SE}(\omega)$ as in Eq. (S2). By combining Eqs. (S1), (S2), (S5), (S7) and (S8), we find that

$$\frac{\tilde{G}^{\mathrm{ref}}(\omega)}{g_{\mathrm{sf}}} = \frac{\tilde{j}_s^{\mathrm{ref}}(\omega)}{\mathrm{i}\omega\tilde{M}(\omega)} = \frac{\tilde{S}^{\mathrm{ref}}(\omega)}{\tilde{S}_M(\omega)} \frac{\tilde{\mu}_s(\omega)}{\Delta\tilde{\mu}_s(\omega)} \frac{2\tilde{Z}_{\mathcal{F}}(\omega)d_{\mathcal{F}}\tilde{n}(\omega)}{\tilde{Z}^{\mathrm{ref}}(\omega)\tilde{\theta}_{\mathrm{SH}}(\omega)\tilde{\lambda}_{\mathrm{rel}}(\omega)c}. \tag{S10}$$

As the third factor in Eq. ($S10$) is constant in the frequency range considered here[3,4], Eq. ($S10$) becomes

$$\frac{\tilde{S}^{\mathrm{ref}}(\omega)}{\tilde{S}_M(\omega)} \propto \frac{\tilde{G}^{\mathrm{ref}}(\omega)}{g_{\mathrm{sf}}} \frac{\Delta\tilde{\mu}_s(\omega)}{\tilde{\mu}_s^{\mathcal{F}}(\omega)}. \tag{S11}$$

Fig. S2a displays the THz signals S_M and S^{ref} in the time domain, and Fig. S2b shows their Fourier amplitude. We observe that the signals have the same dynamics as found previously[4]. Further, Fig. S2c,d displays the amplitude and phase of the ratio $\tilde{R}(\omega) = \tilde{S}^{\mathrm{ref}}(\omega)/\tilde{S}_M(\omega)$, which is constant to very good approximation in the relevant frequency range.

Eq. (S11) and $\tilde{R}(\omega) = \mathrm{const}_\omega$ (Fig. S2c,d) imply (i) $\tilde{G}^{\mathrm{ref}}(\omega) = g_0^{\mathrm{ref}} = \mathrm{const}_\omega$ and (ii) $\Delta\tilde{\mu}_s(\omega) \propto \tilde{\mu}_s^{\mathcal{F}}(\omega)$. In other words, (i) the spin conductance of the CoFeB/Pt interface is frequency-independent, and we find $G^{\mathrm{ref}}(t) \propto \delta_{\mathrm{xp}}(t)$ in the time domain (Fig. S2e). (ii) The spin voltage $\mu_s^{\mathcal{H}}$ of $\mathcal{H}$ = Pt is minor, resulting in Eq. (S5).

Supporting Note 4. Prevalence of spin transport from CoFeB to Pt for $d \leq 6$ Å

The THz signal $S = S_{\mathcal{FH}} + S_{\mathcal{F}}$ from $\mathcal{F}$|MgO(d)|$\mathcal{H}$ stacks has two major contributions $S_{\mathcal{FH}}$ and $S_{\mathcal{F}}$. The component $S_{\mathcal{FH}}$ results from ultrafast spin transport from $\mathcal{F}$ through MgO(d) to $\mathcal{H}$ and subsequent spin-

to-charge conversion in $\mathcal{H}$ = Pt. Any other contribution $S_{\mathcal{F}}$ must stem from layer $\mathcal{F}$ alone, e.g., due to ultrafast demagnetization or odd-in-$\boldsymbol{M}$ photocurrents due to a possible structural inversion asymmetry of $\mathcal{F}$.[4] Importantly, we expect that $S_{\mathcal{F}}$ is independent of the MgO thickness d, whereas $S_{\mathcal{H}}$ strongly decreases with increasing d.

Fig. S1a reveals that the THz-signal amplitude decreases markedly from $d = 0$ to 5 Å. Therefore, these signals are dominated by $S_{\mathcal{FH}}$ and, thus, $j_s(t)$, which permits a reliable extraction of the spin conductance. In contrast, for $d \geq 8$ Å, the amplitude of the THz signals does not change any more (Fig. S1b). Consequently, these signals are dominated by $S_{\mathcal{F}}$, and one cannot extract the spin conductance for these samples.

One could subtract any signal $S|_{d\geq 8\,\text{Å}} \approx S_{\mathcal{F}}$ for $d \geq 8$ Å from the signals $S = S_{\mathcal{FH}} + S_{\mathcal{F}}$ for $d =$ 0-5 Å and, thus, obtain a better approximation to $S_{\mathcal{FH}} \approx S - S|_{d\geq 8\,\text{Å}}$ than $S_{\mathcal{FH}} \approx S$. We confirm, however, that this correction modifies the extracted spin conductance $G_S(t)$ for $d =$ 0-5 Å only negligibly.

Supporting Note 5. Fitting by Eq. (5)

To change the MgO thickness d, the sample needs to be changed. This procedure may result in time-axis shifts of the measured THz emission signal due, e.g., to variations of substrate thickness, which change the light propagation time. To take this effect into account, fitting of the measured spin conductance $G_s(t)$ by Eq. (5) includes a time shift t_0 as free parameter. We find typical values of $|t_0| <$ 10 fs, consistent with previous work[2]. We note that the t_0 variation does not change the fit result for the other parameters because they are greatly decoupled from t_0.

Supporting Note 6. Analysis of A and Eq. (6)

For a MgO thickness $d < 2$ Å, the tunneling current can be comparable to transport at $d = 0$, i.e., $g_0^{\text{CT}} \sim g_0^{\text{ref}}$. However, in this case and for our samples, $f^{\text{PH}} \gg 1 - f^{\text{PH}}$, and Eq. (6) yields $A \approx f^{\text{PH}}$. For larger d, tunneling is significantly less efficient than normal transport due to its exponential decay, i.e., $g_0^{\text{CT}} \ll g_0^{\text{ref}}$. In this case, Eq. (6) yields $A \approx f^{\text{PH}}$, too. In either case, it follows from Eq. (6) that $B/(1-A) \approx g_0^{\text{IRT}}/g_0^{\text{ref}}$.

Supporting Note 7. Rate-equation model of IRT

To model the IRT dynamics of the $\mathcal{F}|\mathcal{X}|\mathcal{H}$ stack, we assume that (i) only one defect located at, say z, is involved in the IRT process of an electron tunneling from $\mathcal{F}$ to $\mathcal{H}$. (ii) Spin transport does not change the spin-voltage dynamics of $\mathcal{F}$ because transport is only small perturbation[4].

To determine the time-domain spin conductance $g_z^\sigma(t)$ of the IRT process of an electron with spin direction $\sigma =\uparrow$ or $\downarrow$, we consider an impulsive chemical potential $\mu^\sigma(t) = a\delta(t)$ for each sort σ in $\mathcal{F}$. More precisely, to avoid infinitely high electron energies, we assume that $a\delta(t)$ is a peak of finite height and area a, but of a nonzero width that is still shorter than the time scale of all relevant processes of the system.

Current by one defect. The quasi-impulsive $\mu^\sigma(t)$ instantaneously populates a defect at z by tunneling, resulting in a defect occupation of $N_0^\sigma(z)$. Subsequently, the occupation $N^\sigma(z,t)$ decays due to tunneling from z to $\mathcal{F}$ or $\mathcal{H}$ with a rate proportional to the instantaneous occupation, i.e.,

$$\partial_t N^\sigma(z,t) = -\Gamma^\sigma(z) N^\sigma(z,t) \qquad \text{(S12)}$$

with $\Gamma^\sigma(z) = \Gamma_{\mathcal{F}}^\sigma(z) + \Gamma_{\mathcal{H}}^\sigma(z)$ and $\partial_t = \partial/\partial t$. Eq. (S12) yields

$$N^\sigma(z,t) = N_0^\sigma(z) \mathrm{e}^{-\Gamma^\sigma(z)t}\Theta(t), \qquad \text{(S13)}$$

where $\Theta(t)$ is the Heaviside step function. The current density of σ electrons from z to $\mathcal{H}$ is $j_{z\mathcal{H}}^\sigma = -\partial_t N^\sigma|_{z\mathcal{H}} = \Gamma_{\mathcal{H}}^\sigma(z) N^\sigma$ and becomes

$$j_{z\mathcal{H}}^\sigma(t) = g_z^\sigma(t) = N_0^\sigma(z)\Gamma_{\mathcal{H}}^\sigma(z)\mathrm{e}^{-\Gamma^\sigma(z)t}\Theta(t), \qquad \text{(S14)}$$

which equals the time-domain spin conductance $g_z^\sigma(t)$ for IRT through MgO by a defect at z.

Summation over defects. To obtain the total current for each σ, we sum over all defects by integration,

$$g^{\mathrm{IRT}\sigma}(t) = \int \mathrm{d}z\, D_{\mathrm{def}}(z) g_z^{\sigma}(t) \coloneqq \langle g_z^{\sigma}(t) \rangle \int \mathrm{d}z\, D_{\mathrm{def}}(z), \tag{S15}$$

where $D_{\mathrm{def}}(z)$ is the defect density. Eq. (S15) also defines the average $\langle \dots \rangle$ over all defects. We assume a homogeneous defect density D_{def0} and that the $N_0^{\sigma}(z)$ and $\Gamma_{\mathcal{H}}^{\sigma}(z)$ depend exponentially on the distance of the defect at z from $\mathcal{F}$ and $\mathcal{H}$, i.e.,

$$N_0^{\sigma}(z) \propto \gamma_{\mathcal{F}\mathcal{X}}^{\sigma} \mathrm{e}^{-z/\lambda}, \qquad \Gamma_{\mathcal{H}}^{\sigma}(z) = \gamma_{\mathcal{X}\mathcal{H}}^{\sigma} \mathrm{e}^{-(d-z)/\lambda}, \tag{S16}$$

where $\gamma_{\mathcal{F}}$ and $\gamma_{\mathcal{H}}$ can be interpreted as attempt rates. We find

$$g^{\mathrm{IRT}\sigma}(t) \propto D_{\mathrm{def}} \gamma_{\mathcal{F}\mathcal{X}}^{\sigma} \gamma_{\mathcal{X}\mathcal{H}}^{\sigma} d \mathrm{e}^{-d/\lambda^{\sigma}} \mathrm{e}^{-\langle \Gamma^{\sigma}(z) \rangle t} \tag{S17}$$

with

$$\langle \Gamma^{\sigma}(z) \rangle = (\gamma_{\mathcal{F}\mathcal{X}}^{\sigma} + \gamma_{\mathcal{X}\mathcal{H}}^{\sigma}) \frac{1 - \mathrm{e}^{-d/\lambda^{\sigma}}}{d/\lambda^{\sigma}}. \tag{S18}$$

Here, we assumed short enough times $|t| \ll 1/\Gamma^{\sigma}(z)$ for which $\mathrm{e}^{-\Gamma^{\sigma}(z)t}$ can be linearized.

Total spin current. To determine the total spin current, we assume charge neutrality on the time scales relevant to our experiment. In frequency space, the response equations $\tilde{j}_{\mathcal{H}}^{\sigma} = \tilde{g}^{\sigma} \Delta\tilde{\mu}^{\sigma}$ and the neutrality condition $\tilde{j}_{\mathcal{H}}^{\uparrow} + \tilde{j}_{\mathcal{H}}^{\downarrow} = 0$ imply a spin current $\tilde{j}_{\mathrm{s}} = \tilde{j}_{\mathcal{H}}^{\uparrow} - \tilde{j}_{\mathcal{H}}^{\downarrow} = \tilde{g} \Delta\tilde{\mu}_{\mathrm{s}}$ with the total spin conductance[4]

$$\frac{1}{\tilde{g}} = \frac{1}{2}\left(\frac{1}{\tilde{g}^{\uparrow}} + \frac{1}{\tilde{g}^{\downarrow}}\right). \tag{S19}$$

Eq. (S19) shows that $\tilde{g}$ is dominated by the lower of the two conductances $\tilde{g}^{\uparrow}$ and $\tilde{g}^{\downarrow}$. This result is plausible: If the initial spin transport is dominated by the more conductive ↑ channel, charge backflow without spin backflow must involve the ↓ channel. Due to this causality chain, $\tilde{g}^{\uparrow}$ and $\tilde{g}^{\downarrow}$ are effectively connected in series, consistent with Eq. (S19), and the total conductance is dominated by the bottleneck of the small $\tilde{g}^{\downarrow}$.

For tunnel transport through MgO, it is known that[5] $\lambda^{\uparrow} > \lambda^{\uparrow}$ and, thus, $\tilde{g}^{\uparrow} > \tilde{g}^{\downarrow}$ at $\omega = 0$. Therefore,

$$g(t) \approx g^{\downarrow}(t), \tag{S20}$$

which relaxes with rate $\langle \Gamma^{\downarrow}(z) \rangle$.

One may argue that the summation over defects [Eq. (S15)] should not be performed before Eq. (S19). However, summation before applying the charge neutrality condition is justified for the following reason. Transfer of a spin from $\mathcal{F}$ to $\mathcal{H}$ causes transient charging and, thus, a transient electric field between $\mathcal{F}$ and $\mathcal{H}$. This field acts on many defects and enables charge backflow through them, not just the one defect through which the initial tunneling event happened.

Impact of interfaces on $\boldsymbol{g^{\mathrm{IRT}}(t)}$. The attempt rates $\gamma_{\mathcal{F}\mathcal{X}}^{\sigma}$ and $\gamma_{\mathcal{X}\mathcal{H}}^{\sigma}$ quantify how efficiently an electron with spin σ is transferred from $\mathcal{F}$ to $\mathcal{X}$ and $\mathcal{X}$ and $\mathcal{F}$, respectively. Therefore, they depend on the properties of the $\mathcal{F}/\mathcal{H}$ and $\mathcal{X}/\mathcal{H}$ interfaces. As a consequence, the interface properties influence the magnitude [Eq. (S17)] and temporal dynamics [Eq. (S18)] of the spin conductance $g^{\mathrm{IRT}}(t)$.

In other words, the spin conductance across a layer $\mathcal{X}$, in principle, not only depends on the bulk properties of $\mathcal{X}$ but also its interfaces. However, the main characteristics of the IRT-based spin conductance of MgO remain unchanged: the spin decay length λ and the increase of the characteristic time τ with the MgO thickness. To gain more insight into the impact of the $\mathcal{F}/\mathcal{H}$ and $\mathcal{X}/\mathcal{H}$ interfaces on $g^{\mathrm{IRT}}(t)$, future studies may measure the THz spin conductance of MgO barriers as a function of the $\mathcal{F}$ and $\mathcal{H}$ material.

References

1. Seifert, T. S.; Jaiswal, S.; Barker, J.; Weber, S. T.; Razdolski, I.; Cramer, J.; Gueckstock, O.; Maehrlein, S. F.; Nadvornik, L.; Watanabe, S.; Ciccarelli, C.; Melnikov, A.; Jakob, G.; Münzenberg, M.; Goennenwein, S. T. B.; Woltersdorf, G.; Rethfeld, B.; Brouwer, P. W.; Wolf, M.; Kläui, M.; Kampfrath, T. *Femtosecond formation dynamics of the spin Seebeck effect revealed by terahertz spectroscopy*. *Nature Communications* **2018,** 9, (1), 2899.
2. Seifert, T. S.; Tran, N. M.; Gueckstock, O.; Rouzegar, S. M.; Nadvornik, L.; Jaiswal, S.; Jakob, G.; Temnov, V. V.; Münzenberg, M.; Wolf, M.; Kläui, M.; Kampfrath, T. *Terahertz spectroscopy for all-optical spintronic characterization of the spin-Hall-effect metals Pt, W and Cu80Ir20*. *Journal of Physics D: Applied Physics* **2018,** 51, (36), 364003.
3. Wahada, M. A.; Şaşıoğlu, E.; Hoppe, W.; Zhou, X.; Deniz, H.; Rouzegar, R.; Kampfrath, T.; Mertig, I.; Parkin, S. S. P.; Woltersdorf, G. *Atomic Scale Control of Spin Current Transmission at Interfaces*. *Nano Letters* **2022,** 22, (9), 3539-3544.
4. Rouzegar, R.; Brandt, L.; Nádvorník, L.; Reiss, D. A.; Chekhov, A. L.; Gueckstock, O.; In, C.; Wolf, M.; Seifert, T. S.; Brouwer, P. W.; Woltersdorf, G.; Kampfrath, T. *Laser-induced terahertz spin transport in magnetic nanostructures arises from the same force as ultrafast demagnetization*. *Physical Review B* **2022,** 106, (14), 144427.
5. Butler, W. H.; Zhang, X. G.; Schulthess, T. C.; MacLaren, J. M. *Spin-dependent tunneling conductance of $\mathrm{Fe}|\mathrm{MgO}|\mathrm{Fe}$ sandwiches*. *Physical Review B* **2001,** 63, (5), 054416.
